# Removal of King's College Hospital

**Published:** 1904-03-19

**Authors:** 


### HOSPITAL ADMINISTRATION.

### CONSTRUCTION AND ECONOMICS.

### REMOVAL OF KING'S COLLEGE HOSPITAL.

APPEAL FOR FUNDS AT THE MANSION HOUSE.

The Lord Mayor (Sir James Ritchie) presided at a great meeting in the Egyptian Hall of the Mansion House on Friday, the 11th inst., in support of the appeal for funds for the removal of King's College Hospital from its present position in Portugal Street, Lincoln's Inn Fields, to a site in South London, selected by a special committee appointed for the purpose, and afterwards purchased and presented to the hospital by the Hon. W. F. D. Smith, M.P.

Not only has King's College Hospital during the 60 years of its existence accomplished a great work in the relief of the sick poor, says the official statement issued, but it has also given many famous men to the service of medicine. Among these are conspicuous in recent times Dr. Todd, Dr. Budd, Dr. Playfair, Sir Wm. Bowman, Sir Wm. Priestley, Sir Alfred Garrod, and Sir Wm. Fergusson, the most renowned operator of his generation. Here the late Sir George Johnson carried out the researches that laid the foundations of our present knowledge of renal diseases; and Lord Lister gained acceptance for the antiseptic methods that have revolutionised modern surgery. The decision to remove was not taken hurriedly. For upwards of a year the question formed the subject of inquiry of various committees appointed expressly for the purpose, and among the principal reasons which led to the conclusion arrived at were the following :—

The condition of the existing building, which, as declared by those best qualified to judge, is not only obsolete in design and inadequate in accommodation, but owing to the limited area of its site, and the method of construction, is incapable of such adequate extension or remodelling as would bring it up to the standard of requirements demanded for a modern hospital.

The removal of the larger number of the poorer inhabitants of the neighbourhood to other and distant parts of London, caused by the clearances in connection with the construction of the new highways from the Strand to Holborn.

The circumstance that the City of Westminster is rich in hospitals, and has recently been further enriched by the enlargement of Charing Cross Hospital, while London as a whole, and particularly South London, is woefully deficient in the provision that is made for its sick poor.

By its removal from a district in which it has become not so greatly required to one in which there is a dense population urgently in need of its many advantages, not only will the hospital be enabled to increase and develop its usefulness, but by its transference the great need of South London will be met without adding to the number of existing hospitals, most of which already have difficulty in meeting their necessary expenditure. On the site of 12 acres on Denmark Hill it is proposed to build a hospital in accordance with the highest requirements of modern science and hygiene. To carry out the scheme and so provide London with a perfectly equipped institution not less than £300,000 is required. In sanctioning a grant of £5,000 by King Edward's Fund towards this sum the Prince of Wales said: "With regard to King's College Hospital, I note with the greatest satisfaction its removal to South London. The importance of establishing in that vast population a large hospital with a medical school is known to all, and I am glad that the decision seems to have met with general approbation."

There was a distinguished gathering both on the platform and in the great hall itself, which was well filled. The meeting throughout was marked by unanimity and enthusiasm.

The Lord Mayor opened the proceedings by saying that they had met to obtain the funds—or at all events to advertise the need of funds—for rebuilding King's College Hospital on the south side of the water where it would be more useful than where it stood at present. A hospital of that description, with Charing Cross Hospital so close at hand, was not now so necessary as when there was a large population in its immediate neighbourhood. Drury Lane and the Strand were being transformed and the people who lived there had been removed. In Camberwell on the other hand, there were large numbers of people very poorly off and not able to provide themselves with the assistance the hospital would be able to afford them. He thought, therefore, the meeting would agree with him that the authorities were wise in their resolution to remove. He had just received what he thought a very pathetic telegram, as well as a practical one. It read: "Lord Mayor London. Sorry unable to attend meeting. As a thank-offering for indoor attention 45 years ago will give 50,000 bricks of own manufacture, carted on to site of new hospital." (Applause.) He did not know how much that represented in cash, but it was certainly a very liberal donation. He had also received a letter from the Bishop of Exeter (Dr. Robertson), formerly Principal of King's College), who deeply regretted that engagements in his diocese prevented his being present to support the movement, which he considered a bold and statesmanlike departure, having regard to the now widening distribution of population, and calculated to secure increased efficiency. "That," added the Lord Mayor, "is the opinion of one whom we all know to be well acquainted with the subject." (Applause.) He called upon the Hon W. F. D. Smith, M.P., chairman of the Joint Committee appointed by the Council of King's College and the Committee of Management of the Hospital.

Mr. Smith (whose rising was greeted with enthusiastic cheers) read the following letter from Archbishop Bourne.

"To my great regret I find, as I feared, that it will not be possible for me to be at the meeting on Friday afternoon. A meeting of Bishops is to take place then and I must be here to receive them and to preside.

I wish your meeting every success as I am sure that the proposed removal of the Hospital to South London deserves every support. The success of your efforts will mean untold advantages to the enormous population on the south side of the Thames. Please express to the meeting my sincere regret for my enforced absence and believe me,

"Yours very sincerely,

"✠ Francis Archbishop of Westminster."

Mr. Meyer the distinguished Free Church Minister in South London had also written a similar letter. Continuing the speaker said he thought he could not do better than give that meeting the reasons which had led the Committee to their decision to move. In the first place they were faced by the unsatisfactory state of the existing buildings, regarding which the sub-committee appointed to inquire into the subject reported last year, that "this Committee having visited the Hospital is of opinion that the construction and arrangements are not adapted to modern requirements; and further that it is not possible to extend on the present site."

No Extension Possible.

The last sentence of that paragraph was the important one. There were many present there who knew that many departments of the hospital were not in so satisfactory a state as they would wish to see them. Modern requirements which had increased very largely in the past few years could not be met on the present site. The Committee considered that it would not be wise, even if it were possible to get it, to appeal for a sum of £60,000 or £100,000 to spend upon a building which, when that amount had been spent, would not be so satisfactory as they would wish one of the chief London hospitals to be.

The Displacement of Population.

The second reason was one to which the Lord Mayor had referred, and that was the comparative failure of the hospital as a local charity. The neighbourhood had greatly changed in the last half century. When the hospital was built, some 60 years ago, there was no district in the whole of London where such an institution was so much needed. The alteration began with the clearance for the Law Courts, and proceeded until it culminated with the clearance for the new street from Holborn to the Strand. To give the meeting some idea of the effect on the population within the area, he might say that that last clearance alone would effect a net reduction of over 4,000 souls. A large number of the people who had been displaced were of the very poorest classes, who most required the presence of a hospital, while the new buildings to be erected would not be of the same kind, but of a far superior nature, and would not require so much hospital accommodation. To take one day for an example: out of 113 in-patients, only 42 were from an area within two miles, and only 10 from the parish in which the hospital was situated. Out of 211 out-patients only 29 were from the parish, and more than 100 came from south of the river, where he hoped the hospital would be built in a few years.

The Effect on the Medical School.

The same conditions affected the efficiency of the medical school. In the last 10 years the maternity cases had dropped from 684 to 389 per annum, and as they all knew, that created a very serious difficulty in regard to the experience to be gained by their medical students. Then the fact that they had to come from so far caused the seriousness of the cases which came to them to decrease, because naturally the more serious cases went to the nearest hospital, and those from a distance did not provide such interesting clinical material as those from shorter distances. If the hospital remained upon the present site they would have to seriously consider in a few years whether it would not be necessary to discontinue the medical school altogether.

No Religious Tests.

There was no foundation for any statement that the decreasing numbers in the medical school was due to the fact that tests were imposed. There were no tests whatever in connection with the hospital, and at the present time there were no tests in connection with the college. That had nothing to do with the decrease in the size of the medical school.

The Need of South London.

The other reason which had influenced the Committee was a more general one, viz. that South London was in great need of a general hospital, and that at present there were proportionately too many hospitals grouped in Central London. These were the four reasons that had influenced them, and he must add that it was with great regret they had felt bound to come to the decision they had. That the question had received careful consideration was shown by the steps they had taken. After it had been dealt with by a joint committee of the General Committee and the Medical Board, a special board was appointed, independent alike of the hospital and the college. They reported that removal was an urgent necessity; and that opinion was finally endorsed by the governors of the hospital, after which a special committee was appointed to arrange for the removal and obtain the necessary funds.

Plans for the New Building.

He thought £300,000 would certainly be required to complete the first instalment of the new buildings. As to the plans, they were proceeding by the method known as limited competition. On the nomination of the President of the Royal Institute of British Architects, Mr. Rowland Plumbe, a gentleman of the highest experience in hospital construction, had consented to act as assessor. They were obtaining plans for a hospital of 600 beds, because though they would not be able to go to that extent at once, unless they were very fortunate, yet they were certain that number would eventually be required. He was glad they had the approval of the King's Hospital Fund, which had given them £5,000, and they had also the complete support of the new neighbourhood in which the hospital was to find its home. They knew that the uprooting of a great hospital from the site on which it had stood for so many years, where it had gained its reputation and accumulated its great traditions, was an onerous undertaking, but they were certain that its traditions would follow it to its new home and that 10 years hence the name of King's College Hospital would be more honoured even than at the present time. (Loud applause.)

The Archbishop of Canterbury rose to move the first resolution. He said that advocacy of a cause whose rightness was so obvious was very difficult, and became almost impossible after such a speech as they had heard from Mr. Smith—the one of all others most entitled to speak on the subject. Summed up, the position was this. They had a great hospital, with fine traditions standing to-day in a condition of inadequacy for its work, just because it was early in the field and so its buildings and appliances were somewhat out of date. That situation was not without its elements of legitimate pride, as indicating the work that must have been done during the half century since those buildings were put up in a region where they were then specially needed. Now their patients were diminishing owing to the lessening of the crowded population of the centre of London. But they would be greatly mistaken if they thought the need was less. The need had transferred itself, and the Hospital must be transferred also. The population of London was increasing year by year and great tracts of crowded streets had arisen south of the Thames, miles away from ready access to hospital accommodation of any kind. Those who worked among them were in despair at the way in which they were left out of touch of hospital aid of which they were in such sore and constant need. To build an additional hospital there would be a splendid plan, but it was impracticable without diminishing the aid afforded to existing institutions. Of course both Guy's and St. Thomas's were actually on the south side of the river but they were within a few yards of it and a hospital was wanted where people needed it most, right in the heart of the new population. He had nothing to add to the eloquence of the facts, but he would like to make a reference to personal grounds for special interest in this matter. He entered upon his work, when he was preparing for ordination, among the poor streets in the region of King's College Hospital—then one of the most crowded and difficult slums in London. The other link was his own association with South London by residence there.

South London an Unknown Region.

South London was a strangely unknown region. He believed that if an examination were held for Fellows of the Royal Geographical Society and they were required to define Horselydown or Nunhead or some other of those districts, for every one who could do it there would be hundreds who could tell them about Greenland's icy mountains and India's coral strand. (Laughter.) South London was one of the least known parts of the British Empire to educated people who read and wrote and thought. In that region was Denmark Hill, where Mr. Ruskin used to live in his childhood. Dickens had described the gardens and lanes of Walworth, which existed not so very long ago. But nowadays that region was so little known just because it was so crowded and uninteresting from the æsthetic point of view and that was just the reason why the hospital was needed there. He rejoiced at the scheme and at the prospect of seeing it fulfilled. He hoped everyone there realised that though no reference was made to the fact in his speech, it was the speaker who had just sat down (Mr. Smith) who was the donor of the site which would make that hospital unique among hospitals anywhere. (Cheers.) The site was some 12 acres, on high ground, and those skilled in statistics could calculate what would be the value of it in a region such as he had described. It would put all London to shame if an adequate response were not made to so generous a gift. (Cheers.) He moved "that this meeting cordially approves the scheme to remove King's College Hospital to South London, and pledges itself to do its utmost to ensure the success of the movement."

Mr. C. E. Tritton, M.P. for the Norwood division of Lambeth, seconded the resolution. He could not say with the Archbishop that South London was an unknown land, for he lived there and had represented part of it in Parliament for the past 12 years. He knew its deficiencies, its difficulties, and its needs, and amongst the chief of these was more hospital accommodation.

The Bishop of Rochester supported the resolution, reminding the meeting that the district to be served by the new hospital extended from Wandsworth to Woolwich, and included Lambeth and Camberwell—ten metropolitan boroughs in all, with over 300,000 people in each, and in all this wide tract of houses hospital accommodation might be said not to exist; their needs and their resources were in inverse ratio to each other.

Dr. Ferrier said that, as representing the medical staff of King's College Hospital, he desired to submit a few words on an aspect of that subject which was worthy of careful attention. Hospitals such as that, though primarily for the sick poor, were just as necessary for the rich and well-to-do; for every case of illness or injury treated in a hospital such as that was an object-lesson to those who in a few years would become the guardians of the public health, and to whom, at one time or another, they would probably have to entrust their own lives or the lives of those nearest and dearest to them. It was therefore of vital importance to the community that those who had to undertake such serious responsibilities should be fully trained and fitted for the important duties they had to perform. It was only in great hospitals which were also educational institutions that that training, that practical experience in the methods of diagnosis and treatment of disease, could be acquired, which they rightly expected on the part of their medical practitioners. King's College Hospital had hitherto deserved well of the public. It had for many years ministered to the sick of a large poverty-stricken district, and it had sent out thousands of men, who were discharging their duties in every part of the globe. It had also been the nursery, if not the cradle, of some of the most important advances of medicine in recent times. Not many years ago Lord Lister was there perfecting and teaching the details of his antiseptic method, which had afforded such benefits to mankind. But medicine did not stand still with the discovery of new methods and new agencies. It was every day increasing in complexity; it was therefore necessary for a hospital such as that to maintain its position in the forefront of progress. The medical and surgical staff had on due consideration unanimously come to the conclusion that it would be in the interests of the hospital and of the medical school that the hospital should be transferred to a more populous district such as that of Camberwell. If they studied their personal feelings and conveniences, they knew it would not be so easy to go from Cavendish Square to Camberwell as to Lincoln's Inn Fields, but such considerations did not weigh with them at all. (Cheers.)

Mr. Watson Cheyne also supported the resolution, which was adopted unanimously amid cheers.

Mr. Charles Awdrey, the treasurer, then read the list of donations, which included : A site of 12 acres, Hon. W. F. D. Smith; Anonymous, £10,000; The Zunz Trustees, £10,000; King Edward's Hospital Fund, £5,000; The Drapers' Company, £5,000; The Ecclesiastical Commissioners, £2,500; W. H. Smith and Son, £2,000; The Merchant Taylors' Company, £1,050; The Clothworkers, £1,000; The Goldsmiths, £1,000; Mr. H. E. Gooch, £1,000; The Mercers Company, Lord Ashton, Lord Burnham, Lord Iveagh, Mr. Awdrey, and Mr. Henry Lloyd, £500 each; Mr. Watson Cheyne, £300; Messrs. Rothschild, £300; Sir Owen Roberts, £250; and Dr. and Mrs. Headlam, £250; sums under £200 (for which they were as grateful as for the larger amounts), £3,412—totalling altogether £106,762.

Lord Methuen (Chairman of the Committee of Management) moved a vote of thanks to the Lord Mayor, to whom he referred as a man having two love affairs on his hands at a time. But the two loves were not rivals, and one of them was quitting him for ever, wishing well to the other —St. Bartholomew's.

Sir John Cockburn seconded, saying that as an old student of the hospital which it now appeared was out of place he rejoiced to assist at the reduction of the dislocation. (Laughter.)

The vote was carried by acclamation, and the meeting separated.

